# Proteomic profile of bone in patients with acromegaly: new insights from a pilot exploratory study

**DOI:** 10.1007/s40618-026-02936-x

**Published:** 2026-06-22

**Authors:** Luigi Demarchis, Sabrina Chiloiro, Pier Paolo Mattogno, Michela Cicchinelli, Penelope Giambò, Federico Valeri, Elena Panizzi, Flavia Angelini, Antonella Giampietro, Emanuele Vodola, Domenico Milardi, Giorgio Quintino D’Alessandris, Laura De Marinis, Liverana Lauretti, Mario Rigante, Antonio Bianchi, Andrea Urbani, Alfredo Pontecorvi, Francesco Doglietto, Federica Iavarone

**Affiliations:** 1 School of Medicine and Surgery, Università Cattolica del Sacro Cuore, Rome, Italy; 2 Department of Medicine, Endocrinology and Diabetology, Fondazione Policlinico Universitario A. Gemelli IRCCS, Largo A.Gemelli, Rome, Italy; 3 Department of Neurosurgery, Fondazione Policlinico Universitario Agostino Gemelli, Istituto Di Ricovero E Cura a Carattere Scientifico (IRCCS), Rome, Italy; 4 Department of Basic Biotechnological Sciences, Intensive and Perioperative Clinics, Fondazione Policlinico Universitario Agostino Gemelli, Istituto Di Ricovero E Cura a Carattere Scientifico (IRCCS), Rome, Italy; 5 Department of Neurosurgery, Mater Olbia Hospital, Olbia, Italy; 6 Otolaryngology and Head-Neck Surgery, Fondazione Policlinico Universitario Agostino Gemelli, Istituto Di Ricovero E Cura a Carattere Scientifico (IRCCS), Rome, Italy

**Keywords:** Acromegaly, Bone, Vertebral fractures, GH, IGF-I, Profilin-1, Periostin, Apolipoprotein E, Caveolin-1

## Abstract

**Introduction:**

Bone metabolism is typically impaired in patients with acromegaly due to increased bone turnover, increased bone resorption, and impaired bone neoformation. The pathogenetic mechanisms underlying skeletal fragility in patients with acromegaly remain not fully clarified. We aim to compare the bone proteome of patients with acromegaly to that of a control group of patients with non-secreting pituitary tumors (NSPTs).

**Methods:**

A Liquid Chromatography-Mass Spectrometry was conducted on ethmoid samples (after processing and digestion of the sample) of five patients with acromegaly and five patients with NSPTs, to identify and assay the proteome. Biological functions were investigated for proteins that were found quantitatively up- and down-regulated in the bone of acromegalic patients, with a ratio of variation based on Fold-Change (FC) >|1.50| and statistical significance (p-value < 0.05).

**Results:**

312 proteins belonging to each group were identified. Six proteins with positive FC (up-regulated) and 12 proteins with negative FC (down-regulated) significantly differ in patients with acromegaly than in patients with NSPTs. Among up- and down-regulated proteins, profilin-1, isoform 5 of the periostin, apolipoprotein E, and caveolin-1 were known to be involved in bone metabolism. In our cohort, a positive correlation was detected between the profilin-1, the isoform 5 of the periostin, GH, and IGF-I levels; and a negative correlation was detected between caveolin-1 and serum GH and IGF-I levels, and with apolipoprotein E and serum IGF-I levels.

**Conclusion:**

Our results proved that the bone of patients with acromegaly is characterized by a specific proteomic profile, which is closely correlated to GH and IGF-I hypersecretion.

## Introduction

Acromegaly is a rare systemic disease, caused by an excessive and autonomous secretion of growth hormone (GH), due in most cases to a GH-secreting tumor of the pituitary gland, and a subsequent hyperproduction of the insulin-like growth factor-1 (IGF-1) [1]. The excess of GH and IGF-I therefore determines systemic comorbidities, such as cardiovascular, respiratory, metabolic, neoplastic, skeletal, and osteoarticular ones [2–4]. Skeletal complications are typically kaleidoscopic in patients with acromegaly, affecting from 30 to 80% of patients and including acral enlargement, facial changes, arthropathy, osteopenia, osteoporosis, and fragility fractures [5]. In particular, most studies agree that patients with acromegaly have an increased risk of vertebral fractures compared to the general population, with a prevalence that varies across cohorts [6–8]. The incidence of fractures remains high even after biochemical control of the disease [8, 9]. In patients with acromegaly, it is well recognized that acromegaly is characterized by high bone turnover, with enhanced bone resorption and impaired bone neoformation [8, 10, 11]. The main feature of this skeletal fragility is alteration of bone microarchitecture, with reduced trabecular bone and increased cortical porosity, despite bone mineral density often being normal or elevated [6–9]. In recent years, many efforts have been made to better understand this particular type of skeletal fragility: most studies published in the literature have been conducted using radiological methods, such as DXA, HR-pQCT, or CBCT, to examine variables such as bone mineral density, trabecular bone score, 3D microarchitecture, and trabecular density [12–16].

Despite the clinical relevance of skeletal fragility progressively increasing in recent years, the underlying pathogenic mechanisms remain not fully clarified in patients with acromegaly.

The histological, molecular, and biochemical study of bone tissue in patients with acromegaly could reveal new elements, not yet known, that could be one of the components leading to the bone damage mechanism characteristic of acromegaly. In recent years, -omic technologies (genomics, proteomics, transcriptomics, metabolomics, etc.), applied to various tissues and biological samples, have provided a greater understanding of the mechanisms underlying various diseases, enabling great strides to be made towards personalized medicine [17–19]. Furthermore, the identification of new molecular targets of skeletal fragility could reduce diagnostic delay and potentially identify novel therapeutic targets for the development of new drugs able to modulate bone metabolism. Therefore, this study aims to identify and analyze the bone proteome in patients with acromegaly.

### Aim of the study

Our study aims to identify and analyze the bone proteome of a group of patients with acromegaly and compare it with that of unaffected patients.

### Patients and methods

A cross-sectional and monocenter study was conducted on patients with GH-secreting pituitary tumor (GHST)/GH-PitNETs and patients with non-secreting pituitary tumors (NSPT)/PitNETs(control group), with the availability of ethmoid bone samples.

Inclusion criteria for the study group were:

1. patients with ascertained diagnosis of acromegaly, according to actual diagnostic criteria [20],
2. patients naïve to GH/IGF-I lowering therapies before surgery;
3. indication to transsphenoidal surgery according to the latest guidelines [21, 22],
4. availability of ethmoid bone samples collected during transsphenoidal surgery for the removal of the GHST-;
5. Agree to participate in the study by signing the informed consent

Inclusion criteria for the control group were:

1. patients with an ascertained diagnosis of non-secreting pituitary tumor;
2. transsphenoidal surgery according to the latest guidelines [23, 24],
3. availability of ethmoid bone samples collected during transsphenoidal surgery for the removal of the non-secreting PitNETs;
4. agree to participate in the study by signing the informed consent.

Exclusion criteria for both groups were:

1. bone invasive PitNET;
2. ascertained diagnosis of primary or secondary osteopenia/osteoporosis;
3. history of fragility fractures;
4. concomitant conditions of insufficient hormone secretion, such as hypothyroidism, hypogonadism, hypercortisolism, and hypoprolactinemia;
5. concomitant conditions of other hormone hypersecretion;
6. concomitant condition associated with osteopenia/osteoporosis;
7. presence of risk factors of osteopenia/osteoporosis;
8. concomitant treatments with drugs known to modulate bone metabolism;
9. concomitant active neoplasia.

Serum GH, IGF-1, calcium, vitamin D, and alkaline phosphatase were measured the day before transsphenoidal surgery. Bone density was assessed using dual-energy X-ray absorptiometry (DXA) at the anteroposterior lumbar spine (L1-L4) and the femoral neck before transsphenoidal surgery.

### Biological sample management and analysis methods

After collecting a sample of ethmoid bone during transsphenoidal surgery for the removal of a /PitNET, protein purification and digestion were performed according to the filter-aided sample preparation (FASP) protocol [25]. For each bone sample, 50 µg (µg) of protein underwent reduction (using 8 mM dithiothreitol -DTT- in urea buffer – 8 M urea and 100 mM Tris), alkylation (with 50 mM iodoacetamide -IAA- in urea buffer), and digestion by trypsin on Microcon Centrifugal Filter Devices (Merck Millipore Ltd., Cork, Ireland) at a final concentration of 1 μg/μL.

For bottom-up proteomic analysis, an UltiMate 3000 RSLC nano – HPLC System (Thermo Fisher Scientific, Waltham, MA, USA) coupled with a high-resolution Orbitrap Fusion Lumos Tribrid Mass Spectrometer (Thermo Fisher Scientific) equipped with a nano-ESI source were used. Peptides were separated on a PepMap RSLC C18 column (2 µM, 100 Å, 50 µm × 15 cm, Thermo Fisher Scientific) using gradient elution. Eluent A consisted of an aqueous solution of 0.1% formic acid (FA), while eluent B was acetonitrile (ACN) with 0.1% FA. The gradient program was as follows (total runtime: 155 min): 3% B and 97% A (min 0–110), 20% B and 80% A (min 110–120), 40% B and 60% A (min 120–125), 90% B and 10% A (min 125–145), 3% B and 97% A (min 145–155), with a flow rate of 0.3 μL/min. Each injection volume was 5 μL (containing a total of 1 μg of peptides), with an NSI ion source type, positive polarity (voltage 1800 V). MS parameters included data-dependent scan mode (DDS) for acquiring high-resolution MS/MS spectra with an Orbitrap detector, a resolution of 120,000 in the 375 – 1500 m/z range, and HCD fragmentation. Samples were analysed in analytical triplicate.

MS/MS data were processed using Proteome Discoverer 3.2 (2025, Thermo Fisher Scientific) based on the SEQUEST HT algorithm (University of Washington, USA, licensed to Thermo Electron Corp., San Jose, CA, USA) against the Uni-ProtKB/Swiss-Prot *Homo sapiens* database. The parameter settings were as follows: minimum precursor mass, 350 Da; maximum precursor mass, 5000 Da; total intensity threshold, 0.0; minimum peak count, 1; signal-to-noise (S/N) threshold, 1.5; precursor mass tolerance, 10 ppm; fragment mass tolerance, 0.02 Da; use average precursor mass, False; use average fragment mass, False; maximum missed cleavage, 2; minimum peptide length, 6; maximum peptide length, 144. Oxidation / + 15.995 Da (M) was set as a dynamic modification, Carbamidomethyl / + 57.021 Da (C) as a static modification, with an FDR rate of 0.01 (Strict) and 0.05 (Relaxed).

Protein abundance was determined through a label-free quantification (LFQ) analysis with the following settings: precursor abundance, Area; protein abundance calculation, Top 3 Average.

The computational normalization strategy Peptide Total Amount was used during protein relative quantification, provided by Proteome Discoverer’s quantification workflow.

The results were filtered for high confidence with ≥ 2 unique peptides.

The bioinformatics analysis was performed using differential expression analysis, with a Fold Change (FC) > 1.5 and p-value < 0.05 as thresholds for statistical significance. FC ratios and t-test p-values were calculated using MATLAB Statistics and Machine Learning Toolbox (R2024a version, MathWorks Inc., Natick, MA, USA). Pathway analysis was performed using the Reactome database (http://reactome.org/). All graphs and heatmaps were generated using MATLAB Statistics and Machine Learning Toolbox (R2024a version, MathWorks Inc., Natick, MA, USA).

## Results

### Clinical and pathological features of the cohort

We collected and analyzed five samples of ethmoid bone from patients with GHST and five samples of ethmoid bone from patients with non-secreting pituitary tumors NSPT. All patients underwent transsphenoidal surgery between January and December 2022. Clinical, morphological, and biochemical data of the study group are shown in Tables 1 and 2. Among patients with acromegaly, 2 were males (40%), and 3 were females (60%); among patients with NSPT, 3 were males (60%), and 2 were females (40% p = 0.5). The median age of the entire study population was 49.5 years (IQR: 19.5), with no significant difference between patients with GHST and those with NSPT (respectively, 49 years, IQR: 31.5, and 55 years, IQR: 22; p = 0.151).

**Table 1:** Clinical data of the study group (patients with GH-secreting pituitary tumor -GHST)

Patients	Gender	Age at diagnosis (years)	Preoperative IGF-1 (ng/mL)	Preoperative GH (ng/mL)
P1	F	21	506	15
P2	F	49	455	3.4
P3	M	59	435	11
P4	M	49	630	9.7
P5	F	24	639	13

GH: growth hormone; IGF-1: insulin-like growth factor-1

**Table 2:** Preoperative bone turnover markers, DXA scores, and electrolytes of the study group (patients with GH-secreting pituitary tumor -GHST)

Patients	Vit D (ng/dL)	Calcium (mg/dL)	AP (UI/L)	DXA TS/ZS L	DXA TS/ZS FN
P1	16	9.4	127	0.4 (ZS)	0.1 (ZS)
P2	22	9.5	80	-1.2 (TS)	-0.8 (TS)
P3	28	8.9	50	3.4 (TS)	0.4 (TS)
P4	80	9.8	62	0.4 (TS)	-0.2 (TS)
P5	30	8.4	60	1.1 (ZS)	0.6 (ZS)

AP: alkali phosphatase; FN: femorale neck; L: lumbar [1-4],TS: T-score,ZS: Z-score

All patients with GHST had laboratory confirmation of acromegaly (median GH: 11 ng/mL IQR: 7.45, and median IGF-I: 506 ng/mL IQR: 189). Pathology examination of the removed PitNET confirmed the diagnosis of somatotropinomas with positive immunohistochemistry for Pit1 and GH. All tumors in the control group (NSPT) were SF1-positive on immunohistochemical examination and were classified as non-secreting gonadotroph tumors.

### Proteins with different expressions

**Fig. 1:**
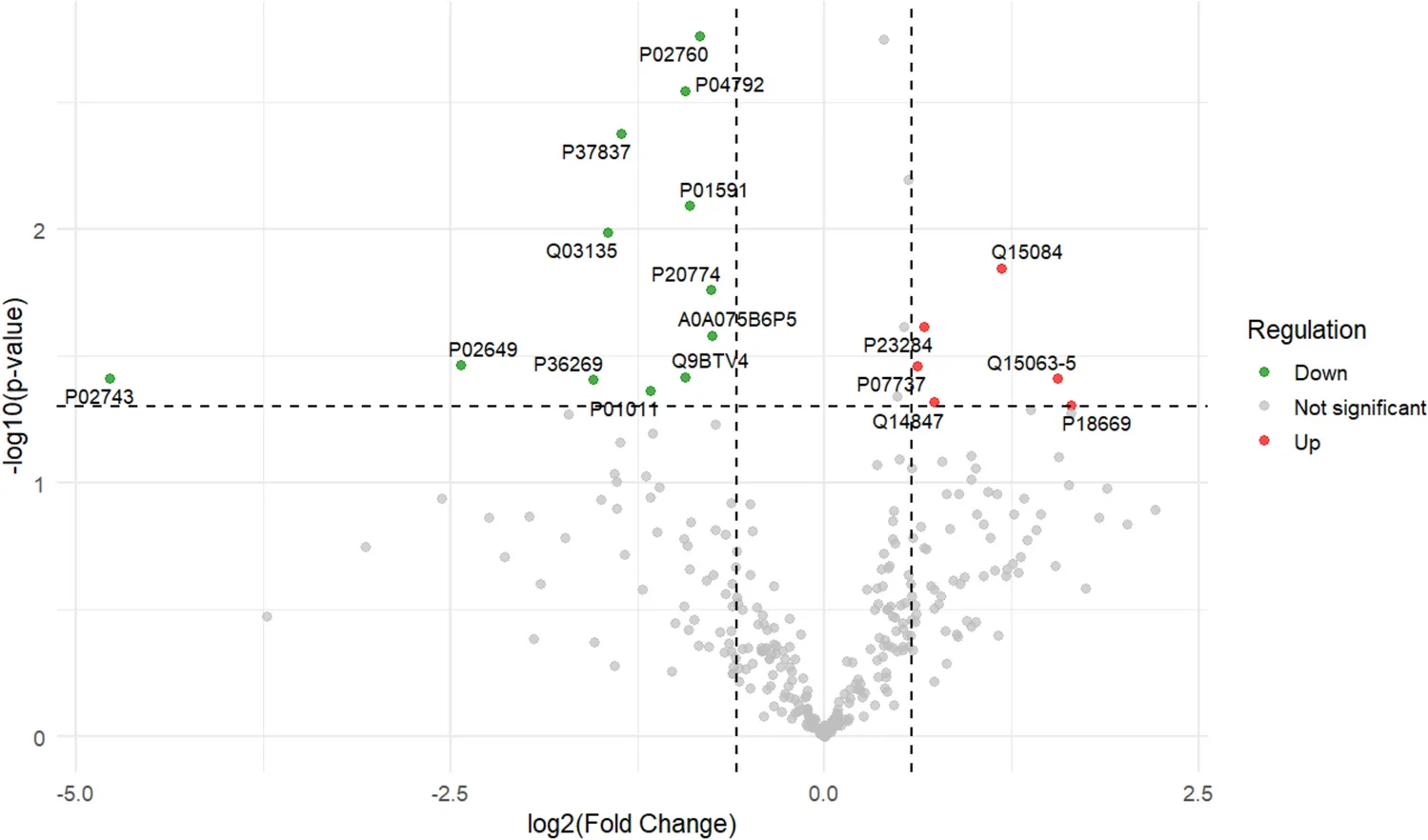
Volcano Plot of the 312 proteins common to the bone of acromegaly and control (non-secreting pituitary tumors NSPT), comparing the ratio between acromegaly (GHPT) and control (NSPT). The parameters used were FC > 1.5 (expressed as Log2FC on the x-axis) and p-value < 0.05 (expressed as -Log10p-value on the y-axis). Proteins significantly upregulated in GHST compared to NSPT are shown in red. Proteins significantly downregulated in GHST compared to NSPT are shown in green

Analysis by LC–MS of the ethmoid samples identified 312 proteins common to both groups. The dispersion of these proteins and their statistical significance between the two groups of patients analyzed are shown in the Volcano plot in Fig. 1. As previously mentioned, we identified proteins that were more expressed (up-regulated) and proteins that were less expressed (down-regulated) in the bone of patients with acromegaly and in the bone of patients with NSPT.

Among the 312 common proteins, 18 resulted in differentially expressed: 6 proteins were up-regulated (IDs: P07737, P23284, Q14847, Q15084, Q15063-5, P18669) and 12 down-regulated (IDs: P02743, P02649, P36269, Q03135, P37837, P01011, P04792, Q9BTV4, P01591, P02760, P20774, A0A075B6P5) in the bone of patients with acromegaly than in the bone of patients with NSPT. As shown in Table 1, the profilin-1, the peptidyl-prolyl cis–trans isomerase B, the LIM and SH3 domain protein 1, the protein disulfide-isomerase A6, the isoform 5 of periostin, and the phosphoglycerate mutase 1 were up-regulated in the bone of patients with acromegaly. Instead, as shown in Table 2, the serum amyloid P-component, the apolipoprotein E, the glutathione hydrolase 5 proenzyme, the caveolin-1, the transaldolase, the alpha-1-antichymotrypsin, the heat shock protein beta-1, the transmembrane protein 43, the immunoglobulin J chain, the protein AMBP, the mimecan, and the immunoglobulin kappa variable 2–28 were down-regulated in the bone of patients with acromegaly.

Each of these 18 proteins was analyzed using String to investigate their biological functions and to identify potential interactions and mechanisms relevant to bone metabolism. Biological correlations with bone metabolism were identified for the profilin-1 and the isoform 5 of the periostin (among the up-regulated proteins) and for the apolipoprotein E and the caveolin-1 (among down-regulated proteins).

**Fig. 2:**
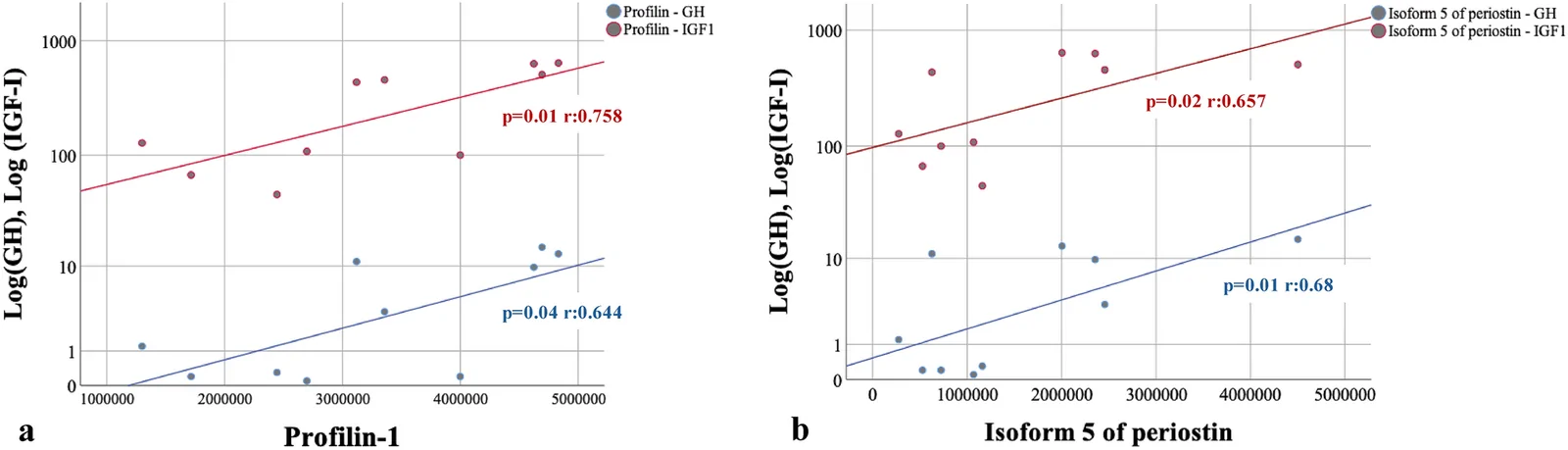
Scatter Plot showing the positive correlation of bone expression of the profillin-1 and serum GH and IGF-I levels (a) and of the isoform 5 of periostin and serum GH and IGF-I levels (b)

**Fig. 3:**
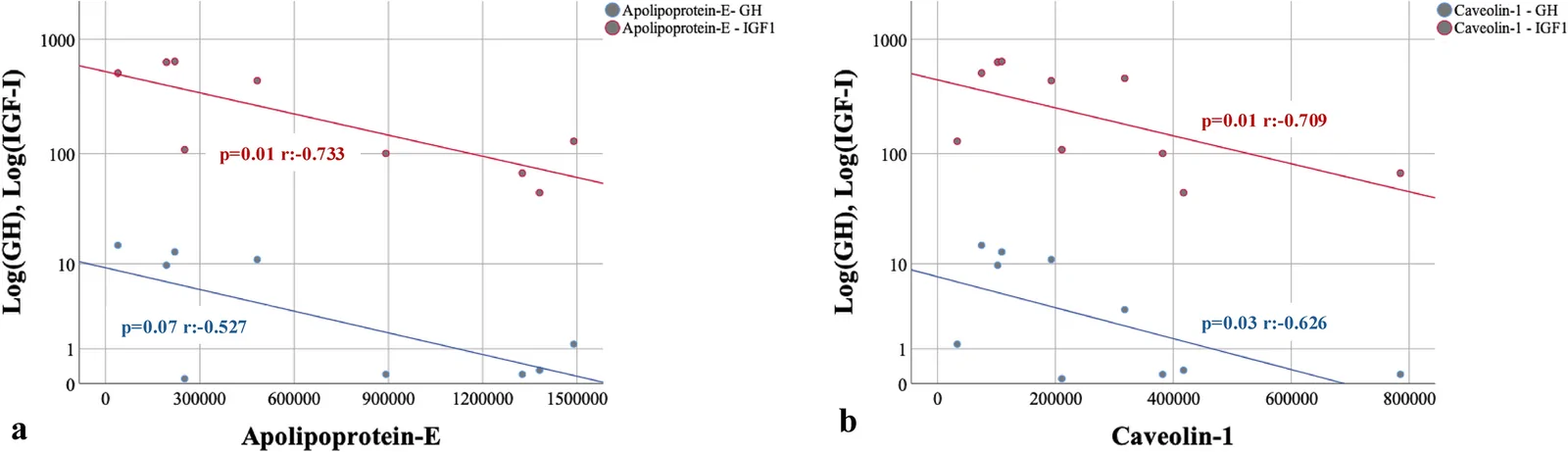
Scatter Plot showing the negative correlation of bone expression of the apolipoprotein-E and serum GH and IGF-I levels (a) and of the caveolin-1 and serum GH and IGF-I levels (b)

A positive correlation was detected between profilin-1 concentration and serum GH (p = 0.04 rho: 0.644) and IGF-I levels (p = 0.011 rho: 0.758). A positive correlation was detected between isoform 5 of the periostin concentration and serum GH (p = 0.01 rho:0.68) and IGF-I levels (p = 0.02 rho: 0.657), as shown in Fig. 2a and b. In parallel, a negative correlation was detected between apolipoprotein E concentration and serum IGF-I levels (p = 0.01 rho: -0.733) and serum GH levels (p = 0.007 rho: -0.527), as shown in Fig. 3a. In addition, caveolin-1 concentration negatively correlated with serum GH (p = 0.03 rho: -0.626) and IGF-I levels (p = 0.01 rho: -0.709), as shown in Fig. 3a.

The membership pathways of the 18 proteins were researched on the Reactome database, and their correlations with bone metabolism in the literature were learned, as shown in Table 5.

**Table 3:** Description of proteins that resulted up-regulated in bone of acromegaly patients compared to controls (patients with non-secreting pituitary tumors -NSPT)

Protein	Protein ID	p-value	Fold change
Profilin-1	P07737	0.03	1.534604
Peptidyl-prolyl cis–trans isomerase B	P23284	0.02	1.582455
LIM and SH3 domain protein 1	Q14847	0.05	1.656822
Protein disulfide-isomerase A6	Q15084	0.01	2.268267
Isoform 5 of Periostin	Q15063-5	0.04	2.934666
Phosphoglycerate mutase 1	P18669	0.05	3.130287

## Discussion

Acromegaly causes profound bone changes due to excess GH and IGF-1, leading to accelerated bone remodeling and increased skeletal fragility compared to the healthy population: patients have a higher risk of vertebral fractures than the healthy population, even with normal or increased bone mineral density (BMD) [6, 8]. Bone formation and resorption are increased in patients with acromegaly, with elevated biochemical markers compared to healthy individuals [6, 8, 11]. An interesting study by Belaya et al. shows that in active acromegaly, bone tissue exhibits few changes in genes characteristic of remodeling but significant changes in microRNAs and TWIST1, suggesting a reduced capacity to form healthy bone and an altered fate of stem cells [26]. Bone formation, however, is typically impaired, resulting in thickened cortical bone with poor quality and poor resistance [15].

To our knowledge, we investigated, for the first time, in vivo the differences in bone proteome expression in patients with acromegaly in comparison with a control group of patients with NSPT, using ethmoid bone samples. We identified 18 proteins with different expressions in patients with acromegaly compared to controls: 12 proteins were less expressed, and 6 proteins were more expressed in patients with acromegaly than in controls (Tables 1 and 2).

The profilin-1 and the isoform 5 of the periostin (among the up-regulated proteins) and apolipoprotein E and the caveolin-1 (among the down-regulated proteins) have already been reported in several studies on bone metabolism, not yet in acromegaly.

Profilin-1 (PFN1) plays a key role in the cell cytoskeleton, binding to actin monomers and regulating their polymerization [27, 28]. The functions of PFN1 are not yet entirely clarified, although an inhibitory role in osteoclast migration has been observed, enhancing bone resorption at least [29]. This mechanism is consistent with the pathological metabolism of cortical bone in patients with acromegaly, due to the reduction in bone turnover and osteoclastic activity, as opposed to that observed in trabecular bone [30, 31]. PFN1 is also expressed in osteocytes, and its reduction results in decreased bone volume and mineral density [32]. The PFN1 deficiency also leads to delayed formation of endochondral bone and particularly long bones [33]. Wu et al. proved that *PFN1* gene polymorphisms were associated with bone mineral density and osteoporotic fractures in Chinese males [34]. The loss-of-function of profilin-1 for deletions and missense mutations of the *PFN1* gene has been identified in families with early-onset Paget bone disease and giant cell tumors [35, 36]. To date, no studies have reported the interactions between PFN1 and osteocytes in patients with acromegaly. However, it has been proven that bone fragility in patients with acromegaly is also due to increased turnover and osteoblastic dysfunction [37]. Some molecules, such as sclerostin, are already known to interact with these cells, like osteocytes, in the regulation of bone turnover [30, 38].

Periostin (POSTN), also known as osteoblast-specific factor 2 (OSF2), is a protein particularly expressed in bones and in fibrous connective tissues with high collagen concentration [39]. Periostin acts as a scaffold for the assembly of extracellular matrix proteins and accessory proteins [40]. Besides being a structural molecule of the bone matrix, periostin also acts as a signaling molecule through integrin receptors and Wnt-beta-catenin pathways, to stimulate osteoblast functions and bone formation [41]. Several isoforms of periostin have been identified, but their specific functions remain largely unknown to date [42]. Periostin is involved in several mechanisms of bone metabolism. Circulating periostin was recently suggested as a novel biomarker of bone health, in particular in conditions of increased bone turnover [43]. Elevated serum periostin levels were reported in patients with hyperparathyroidism and in patients with type 2 diabetes mellitus, and inversely correlated with BMD [44, 45]. Serum periostin levels also increased during therapy with teriparatide [46]. An interesting study by Robubi et coauthors, conducted on cultured human osteoblasts in vitro treated with different growth factors, showed that IGF-1 results in increased expression of several genes, including POSTN [47]. IGF-1 and periostin are involved in the regulation of the periosteal surface of cortical bone [41, 48]. Serum periostin levels positively correlated with IGF-I levels, resulting in more elevated levels at the end of the growth (16–18 years) than at the peak of bone mass and at advanced age, also reflecting the role of IGF-I in bone cortical consolidation and remodeling [49]. The results of our study are consistent with previous findings: as in our patients with acromegaly, periostin was found to be upregulated. Interestingly, the results of our study proved that the bone expression of the up-regulated proteins (profilin-1 and isoform 5 of the periostin) positively correlated with serum GH and IGF-I levels, suggesting a direct interaction between all these molecules.

Apolipoprotein E (ApoE) is mainly synthesized in the liver and plays a key role in lipid metabolism [50]. Recently, several studies have also highlighted its role in regulating bone health: ApoE promotes osteoblast differentiation and inhibits osteoclast formation [51]. ApoE deficiency leads to a loss of bone mass [52]. Some polymorphisms, particularly ApoE4, may increase the risk of osteoporosis and fractures [53], however, the results are not always consistent across different populations [54]. Caveolin-1 is a highly ubiquitous protein found in many tissues throughout the human body. Caveolin-1 is involved in cell membrane maintenance, cellular signaling, differentiation, proliferation, and migration, as well as regulation of programmed cell death and autophagy [55, 56]. In a study conducted in mouse models by Lee et al., it was proved that caveolin-1 plays a complex role in the regulation of osteoclastogenesis and bone metabolism, with sex-dependent effects: in female mice, the lack of caveolin-1 leads to an increase in bone volume and a decrease in osteoclasts, while in male mice, the lack of caveolin-1 leads to increase in both osteoclasts and osteoblasts, without changing the final bone volume [57]. Jia et al. observed in a mouse model that the inhibition of Caveolin-1 promotes bone formation through an angiogenic mechanism [58]. In our study, Caveolin-1 and ApoE were down-regulated in patients with acromegaly compared to the control group, providing also a negative correlation between their bone expression and serum GH and/or IGF-I levels, suggesting a direct interaction between all these molecules.

Mimecan and Heat shock protein beta-1 were also found to be down-regulated in patients with acromegaly in our study. Mimecan, also known as osteoglycine, is a proteoglycan that contributes to the organization of collagen fibrils [59]. There are currently no studies that describe a direct function of mimecan on bone metabolism. Heat shock protein beta-1 is a chaperone protein of the heat shock protein family and is involved in the cellular response to stress and regulation of proteostasis [60]. Although its inherent functions in bone metabolism remain unclear, several studies suggested an involvement in bone formation and resorption, regulating the activities of osteoclasts and osteoblasts [61–64].

Another interesting, statistically significant finding is the concentration of serum amyloid P-component in this study: the FC value of this protein is significantly reduced in the group of patients with GHST compared to patients with NSPT. Serum amyloid P component (SAP or also called pentraxin-2) is a pentameric human plasma glycoprotein, produced mainly by the liver, and is involved in the modulation of cell activity, in the removal of necrotic material, and in the regulation of fibrosis and inflammation [65]. It is involved in neurodegenerative diseases and amyloidosis [65–68]. A recent study conducted on mouse models has shown that the accumulation of senescent bone marrow adipocytes leads to an increased SAP secretion, resulting in the formation of amyloid deposits in the bone marrow and bone loss [69].

Protein Disulfide Isomerase Family A Member 6 (PDIA6) is a member of the disulfide isomerase (PDI) family of the eukaryotic endoplasmic reticulum and is involved in redox reactions [70, 71]. PDIA6 also acts as a chaperone, contributing significantly to protein folding and maintaining the redox homeostasis in the endoplasmic reticulum [72]. Although the functions of PDIA6 are still not entirely clear, some studies reported its expression or alterations in neoplastic diseases [73, 74]. No studies have proved a notable function or correlation of PDIA6 with osteo-metabolic diseases. Herr et coauthors highlighted that the silencing of *PDIA6* gene led to a reduction in the tumor volume of giant cell neoplasms of bone stromal cells [75]. Furthermore, PDIA6 is involved in the IRE1-XBP1 pathway, which is part of the UPR (Unfolded Protein Response): this protein promotes the inactivation of IRE1alpha [76], and in our study, we found increased expression of PDIA6 in the ethmoid bone of patients with acromegaly compared to the control group. Inhibition of IRE1alpha/XBP1 reduces osteoclast formation and increases bone mass in mouse models [77, 78]. Some studies suggest that the activation of IRE1-XBP1 in mesenchymal cells stimulates the Osx transcription [78, 79], a transcription factor crucial for bone formation [80].

The main limitations of this study are the small number of bone samples collected and analyzed, and the choice of ethmoidal bones for proteomic studies, which does not mirror the typical trabecular structure of the vertebrae [81, 82], reflecting the difficulty in obtaining freshly collected trabecular bone or vertebral samples. Although our cohort includes an equal number of males and females, the sample size—both in the study group and the control group— was too small to allow for stratification of the results by gender. At least, another limitation of this study is the lack of dosage of serum concentration of up- and down-regulated proteins, which may also suggest whether these molecules may be considered as novel markers of bone metabolism activity in patients with acromegaly. However, the results of our study showed, for the first time to our knowledge, the up- and downregulation of specific proteins in the bone of patients with acromegaly, such as profilin-1, isoform 5 of the periostin, apolipoprotein E, caveolin-1, and a strong association between their expression and circulating GH and IGF-I levels. Although the evidence on the functions of these proteins on bone metabolism is quite limited, our results are promising for the potential identification of novel molecular pathways involved in skeletal fragility in patients with acromegaly. Even though it is clear that patients with acromegaly have an increased risk of vertebral fractures compared to the general population, there are currently no validated markers available that indicate bone damage or predict fracture risk. This first pilot study aims to investigate the bone proteome in patients with acromegaly in order to better understand the mechanisms that expose these patients to a higher risk of fractures. Further prospective studies are advocated to investigate circulating levels of these up- and down-regulated proteins and to investigate their potential role in predicting skeletal fragility and the risk of fractures in patients with acromegaly.

**Table 4:** Description of proteins that were down-regulated in the bone of acromegaly patients compared to controls (patients with non-secreting pituitary tumors -NSPT)

Protein	Protein ID	p-value	Fold change
Serum amyloid P-component	P02743	0.04	-27.4345
Apolipoprotein E	P02649	0.03	-5.40389
Glutathione hydrolase 5 proenzyme	P36269	0.04	-2.91951
Caveolin-1	Q03135	0.01	-2.73558
Transaldolase	P37837	< 0.01	-2.56364
Alpha-1-antichymotrypsin	P01011	0.04	-2.24336
Heat shock protein beta-1	P04792	< 0.01	-1.91229
Transmembrane protein 43	Q9BTV4	0.04	-1.90901
Immunoglobulin J chain	P01591	< 0.01	-1.87292
Protein AMBP	P02760	< 0.01	-1.78884
Mimecan	P20774	0.02	-1.69367
Immunoglobulin kappa variable 2–28	A0A075B6P5	0.03	-1.68185

**Table 5:** Description of pathways of proteins that were found up-regulated and down-regulated in acromegaly bone compared to controls (patients with non-secreting pituitary tumors -NSPT)

Pathways of proteins up-regulated in acromegaly bone	Entities found	Entities total	p-value
P130CAS Linkage to mapk signaling for integrins	3	15	< 0.001
GRB2: SOS Provides linkage to mapk signaling for integrins	3	15	< 0.001
MYD88 deficiency (TLR2/4)	3	19	< 0.001
IRAK4 deficiency (TLR2/4)	3	20	< 0.001
Regulation of tlr by endogenous ligand	3	21	< 0.001
Pathways of proteins down-regulated in acromegaly bone			
Extracellular matrix organization	3	300	< 0.001
Acrosome reaction and sperm:oocyte membrane binding	1	6	0.03
Uptake and function of diphtheria toxin	1	7	0.003
Defective B4GALT1 causes B4GALT1-CDG (CDG-2D)	1	8	0.003
Defective ST3GAL3 Causes MCT12 AND EIEE15	1	8	0.003

In conclusion, our proteomic analysis highlighted specific proteins with different expressions in the bones of patients with acromegaly, which could suggest pathophysiological mechanisms of skeletal fragility, as a typical acromegaly-related complication.

## Data Availability

Datasets generated and analyzed during the current study are not publicly available but are available from the corresponding author on reasonable request.

## Abbreviations

ApoE: Apolipoprotein E
BMD: Bone mineral density
Cav1: Caveolin-1
CBCT: Cone beam computed tomography
DXA: Dual-energy X-ray absorptiometry
GH: Growth hormone
GHST: Growth hormone-secreting tumor
HR-pQCT: High-resolution peripheral quantitative computed tomography
IGF-1: Insulin-like growth factor-1
IRE1-XBP1: Inositol-requiring kinase 1—X-box- binding protein-1
LC-MS: Liquid chromatography-mass spectrometry
NSPT: Non-secreting pituitary tumor
OSF2: Osteoblast-specific factor 2
PDIA6: Disulfiride isomerase family a member 6
PFN1: Profilin-1
POSTN: Periostin
SAP: Serum amyloid P component
TS: T-score
UPR: Unfolded protein response
ZS: Z-score
